# Possible Migration and Histopathological Analysis of Injections of Polymethylmethacrylate in Wistar Rats

**DOI:** 10.5402/2012/609158

**Published:** 2012-05-30

**Authors:** Rodrigo d'Eça Neves, Marcello Alberton Herdt, Felipe Barbieri Wohlgemuth, Jorge Bins Ely, Zulmar Antonio Accioli de Vasconcellos, José Caldeira Ferreira Bastos, Armando José d'Acampora

**Affiliations:** ^1^Department of Surgery, Federal University of Santa Catarina (UFSC), 88040-900 Florianópolis, SC, Brazil; ^2^Plastic Surgery Service, University Hospital of the Federal University of Santa Catarina, 88040-900 Florianópolis, SC, Brazil; ^3^General Surgery Program, Hospital Governador Celso Ramos, 88015-270 Florianópolis, SC, Brazil; ^4^Institute of Pathological Anatomy Diagnositcs, Florianópolis, SC, Brazil

## Abstract

*Objective*. To evaluate the possible migration of polymethylmethacrylate after injections in various corporal compartments of Wistar rats. *Methods*. The experimental work consisted in the injection of PMMA in corporal compartments for later histopathological analysis of the locations of implants and of distant filtering organs. The dose applied in each implant was of 0.2 mL. The animals were divided into groups according to the location of the implant realized: group GB had intradermic injections in the glabella. Group SD had subdermal injections in dorsal subcutaneous tissue cells. Group IP had intraperitoneal injections in the abdomen. Group PD had intramuscular injections in the right rear leg. The rats were sacrificed 30 days after realization of the implants and tissue samples from the lung, liver, spleen, and kidney, and locations of implantation were removed for histopathological analysis. *Results*. Characteristic microspheres that were compatible with the presence of PMMA in any of the histological slides analyzed were not observed. One animal had an amorphous exogenous substance, with a histiocytic reaction. Twelve of the 16 lungs analyzed had locations of intraalveolar hemorrhaging. Two animals had nonspecific spleen alterations. *Conclusion*. The histopathological analysis of this study found no PMMA microspheres in any of the tissues analyzed.

## 1. Introduction

In recent years for either aesthetic or reconstructive purposes, a diversity of materials has been used as dermal fillers. The objective is to elevate the area treated by decreasing the thickness of the skin in this region and by improving its appearance [1]. 

Despite the large number of publications about this issue, the ideal substance for filling has still not been found, because the material must be safe, permanent, easy to apply, and have a good cost-benefit ratio and a low rate of complications [2–6]. 

Inert materials tested for injectable microimplants include polymerized silicon, which began to be used in 1940, [4] with the high risk of complications. Others such as hyaluronic acid, fat grafts, and collagen are substances quite commonly used today, but whose results have limited duration [7]. 

PMMA was discovered and synthesized in 1902, by Otto Röhm, and was patented as Plexiglass in 1928. It has been used in medicine since 1945 and now has a variety of applications including bone cement and others [2]. 

In 1991, Gottfried Lemperle began to study polymethylmethacrylate (PMMA) to fill in small skin deformities.

Lemperle et al. showed that PMMA was histological biocompatible and did not generate inflammatory responses when implanted under the skin [8]. Artecoll, a substance to be used as a dermal filler, was created by Rofil Medical International. The product consists of microspheres of PMMA with diameters from 30 to 40 *μ*m, in a bovine colloid vehicle. It was thought to be a solution for a dermal filler, with rare collateral effects and excellent long-term results [9]. Nevertheless, with the increased popularity of its use, there was an increase in the number of publications about its complications.

**Figure 1:**
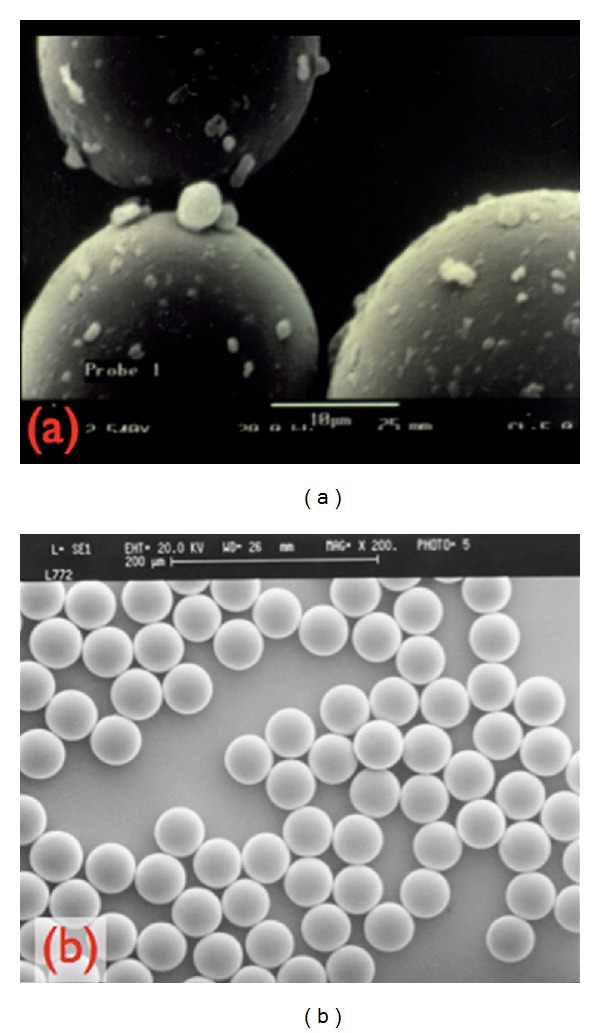
Photos of PMMA under electronic microscope (×800). (a) Microspheres of PMMA of different sizes with impurities in bone cement. (b) Artefill after washing and purification of the PMMA microspheres. source: Lemperle, 2003.

According to Lemperle, these complications were due to impurities that adhered to the microspheres. In 1994, the Artecoll microspheres were modified, so that they would have absolutely smooth and spherical surfaces (Figure 1) [10]. In an attempt to decrease complications even more, new techniques for the removal of impurities were utilized.

In 2006, a third generation was presented, called ArteFill, with rigorous control in the purification of the microspheres now with diameters of 30–50 *μ*m [11]. 

PMMA skin implants are becoming popular and known as bioplasty. An increasing number of doctors from a variety of specialties are becoming trained to offer bioplasty as part of the services provided in their private practices [4]. Nevertheless, the procedures are not completely free of risk, and a number of complications are related to the injection of PMMA.

In Brazil, one of PMMA products is Metacrill (Nutricel, RJ), which has been used for more than 8 years, and 30% of its composition is PMMA microspheres with diameters 40–60 *μ*m, in carboxymethyl-cellyulose coloid [12]. 

The literature about studies of migrations, inflammatory response, composition, and safety is related to the Dutch substance—Artecoll, which has a different composition.

The objective of this study was to observe, through histopathological analysis, the possibility of the migration of polymethylmetacrylate, after injections in various body compartments of Wistar rats.

## 2. Methods

This study was submited to the Commission of Ethics in the Use of Animals of the Federal University of Santa Catarina (UFSC) on June 20, 2007, and was approved on August 15, 2007, according to protocol PP00126.

The experiment utilized 16 adult female Wister rats (*Rattus norvegicus albinus, *Rodentia : Mamalia), produced at UFSC's central breeding facility. The rats were 180 days old and had an average weight of 300 grams.

The experimental study was conducted at the Laboratory of Operating Technique and Experimental Surgery of the Surgery Department of the Federal University at Santa Catarina (TOCE-UFSC), where animals were maintained individually in polyurethane cages of 40 × 32 × 16 centimeters, under ambient temperature and lighting, maintaining the day-night cycle and access to rations and water *ad libitum*. The cages were cleaned every 48 hours, with replacement of water and rations. The rats were observed for the presence of strange behavior, signs of infection, and injuries caused by mutilation, at six-hour intervals during the day, for the entire experiment.

The experimental group (*n* = 16) was divided into 4 sub-groups, each with 4 animals, which were individually selected through a simple drawing and separated into groups according to the location of the implant (Table 1).

### 2.1. Glabella Subgroup (Subgroup GB *n* = 4)

**Figure 2:**
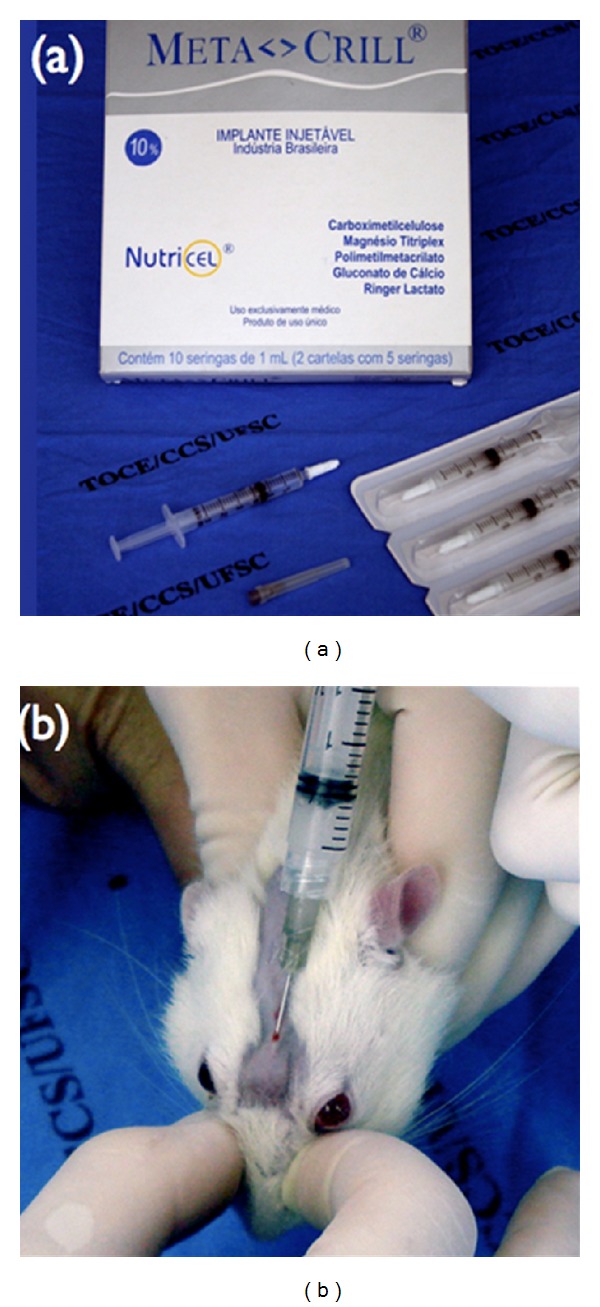
Metacrill. (a) Material used in the study. (b) Injection of 0.2 mL of intradermal polymethylmetacrylate in the glabella region.

 The GB subgroup included four animals which, after receiving anaesthesia, were injected with 0.2 mL of PMMA in the glabella region on the intradermal plane (Figure 2(b)). The animals were submited to painless death induced 30 days after the PMMA injection. For the histopathological procedure of this group, the right eyeball of each animal was removed.

### 2.2. Subdermal Subgroup (Subgroup SD *n* = 4)

**Figure 3:**
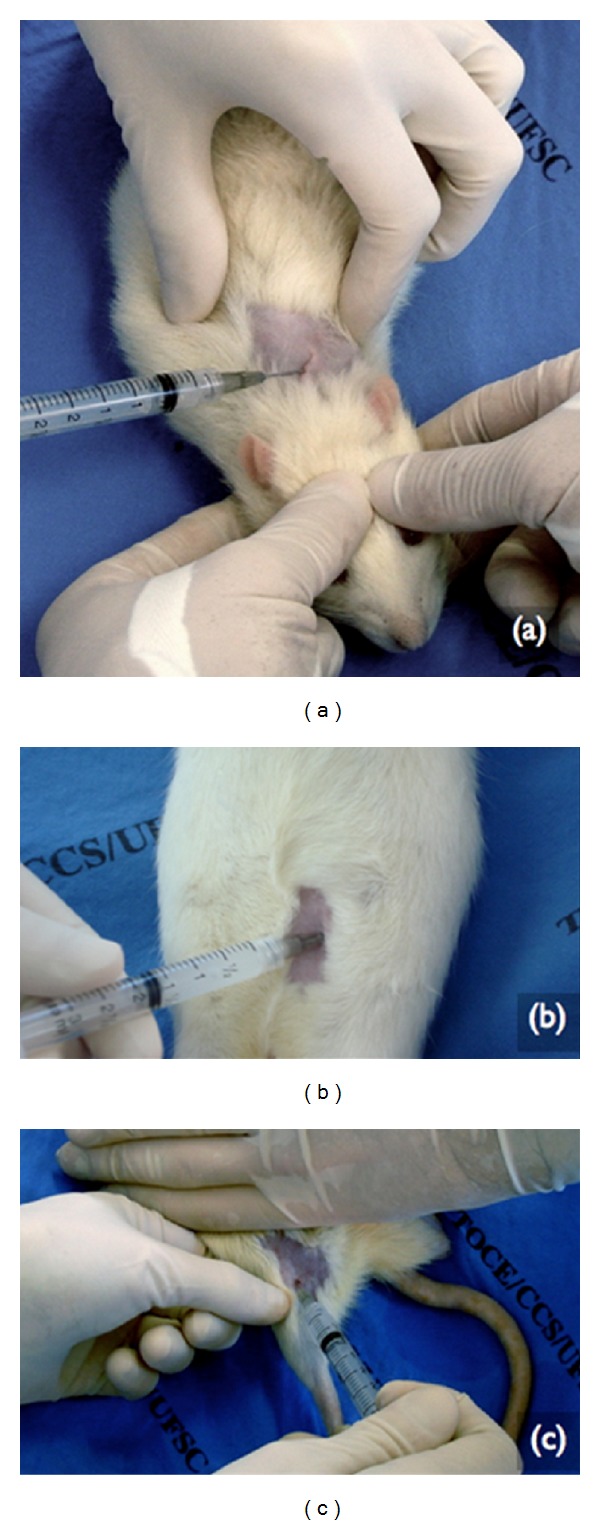
Subdermal application of PMMA in the dorsal (a), intraperitoneal in the right hemi-abdominal (b), and intramuscularly in the right *membrum pelvinum* (c).

 The SD subgroup included four animals which, after receiving anesthesia, were injected with 0.2 mL of PMMA in the cervical dorsal region on the subdermal plane (Figure 3(a)). These animals were submited to a painless induced death 30 days after the PMMA injection. For the histopathological procedure of this group, the skin and subcutaneous cellular tissue were removed from the implanted area of each animal.

### 2.3. Intraperitoneal Subgroup (Subgroup IP *n* = 4)

 The IP sub-group included four animals which, after receiving anesthesia, received injections of 0.2 mL of PMMA in the right hemiabdominal region on the intraperitoneal plane (Figure 3(b)). These animals were submited to painless induced death 30 days after the injection of PMMA. For the histopathological procedure of this group, each animal's diaphragm muscle was removed.

### 2.4. Right Membrum Pelvinum Subgroup (MPD Subgroup *n* = 4)

The MPD subgroup included four animals which, after receiving anesthesia, received injections of 0.2 mL of PMMA in the rear right leg on the intramuscular plane (Figure 3(c)). These animals were submitted to a painless induced death 30 days after the PMMA injection. For the histopathological procedure of this group, the musculature of the right* Membrum pelvinum* of each animal was removed.

### 2.5. Procedures

The anesthesia was applied through an intramuscular injection of atropine, at a dose of 0.044 milligrams per kilo of live weight. Ten minutes later, they were injected with xylazine chlorohydrate at a dose of five mg/kg of live weight and ketamine chlorhydrate at a dose of 10 mg/kg of live weight, both from the same syringe and applied intramuscularly to the inner left thigh. The absence of reflex movements, in response to pinching the *membrum pelvinum*, was the method used to evaluate the effectiveness of the anesthesia. After confirmation of the proper anesthetic effect and the removal of fur from the referred to locations, the animals were submited to the PMMA injection procedures.

All the animals received implants of PMMA of the Metacrill 10% (Figure 3(a)). According to information from the manufacturer, it is composed of polymethylmetacrylate, with a diameter of 40–60 *μ*m, in a colloid medium of carboxy glutonate hydrolactate of magnesium.

The dose utilized, in all cases, was 0.2 mL of Metacrill per animal, using a 3 mL polyurethane syringe and a 25/7 G hypodermic needle.

All the animals were given anesthesia once again, with the technique already described and submitted to a painless induced death through inhalation of ethyl ether in a closed chamber, and, after death, the following organs were removed from all of the animals: right kidney, lungs, liver, and spleen.

For the analysis of local migration, specific organs were removed depending on the location of the injection. In group GB, the entire eyeball was removed with part of the attached optical muscles and nerve. In the animals of the SD group, the skin with the subcutaneous cell tissue from the implanted area was removed. In the IP and MPD groups, respectively, the diaphragm and musculature of the rear right leg were removed (Figure 5). These parts were then set in formaldehyde and placed in recipients identified by numbers corresponding to each rat and respective group. The histopathological analysis was conducted at the Instituto de Diagnóstico Anátomo Patológico (Institute of Pathological Anatomy Diagnostics) (IDAP), in the city of Florianópolis, by a single pathologist.

At the IDAP, since no nodulations or any alteration in the macroscopy of the parts were found, a small random part of the tissue was removed and included in paraffin, cut with a manual microtome at a thickness of four micons. At each cut, a morphometric microscopic, anatomical-pathological analysis was conducted to verify the possible presence of a material compatible with microspheres of injected PMMA. An effort was made to analyze the following cell types:

- polymorphonuclear cells (PMN),
- mononuclear cells (MO),
- foreign body giant Cells (GC),
- macrophages,
- other cell types,
- microspheres.

A total of 80 histological sections colored with hematoxylin/eosin were observed by a single medical pathologist, using an optical microscope.

## 3. Results

### 3.1. Group GB

**Figure 4:**
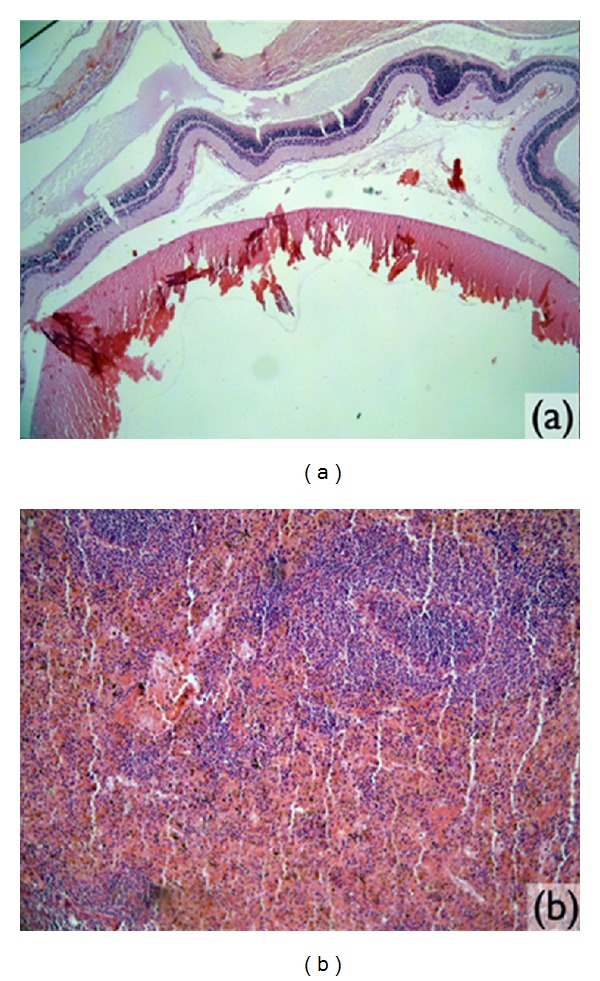
Microscopic images observed in the study: (a) right eyeball (magnified 4x) and (b) spleen tissue (magnified 4x).

In the histological sections of the eyeball of the four animals studied, all the visual structures, the optical nerve, sclera, retina, cornea, and crystalline lens, did not present any modifications to the morphological standard or any inflammatory infiltration (Figure 4(a)).

 The sections of the spleen analyzed had normal histological standards with white and red pulp and no significant alterations (Figure 4(b)).

**Figure 5:**
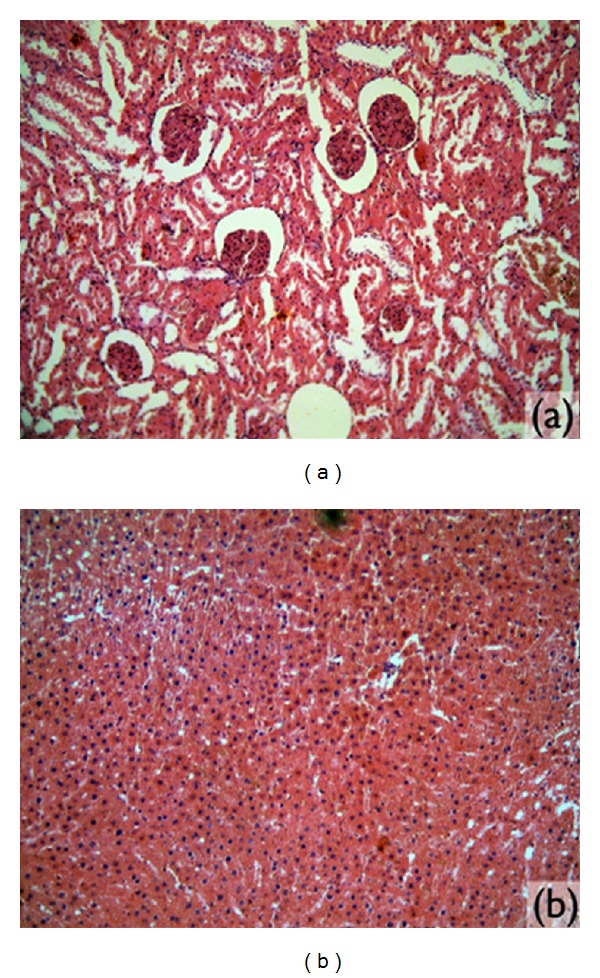
Microscopic images. (a) Kidney tissue analyzed (magnified 10x). (b) Liver tissue (magnified 10x).

The histological sections of the renal tissue revealed blood vessels, glomerulus, tubules, and interstice without significant alterations (Figure 5(a)). The liver had normal morphology, with parenchyma of trophic lobules and an absence of inflammatory infiltrations (Figure 5(b)).

None of the sections presented material with characteristics similar with microspheres or giant macrophages.

**Figure 6:**
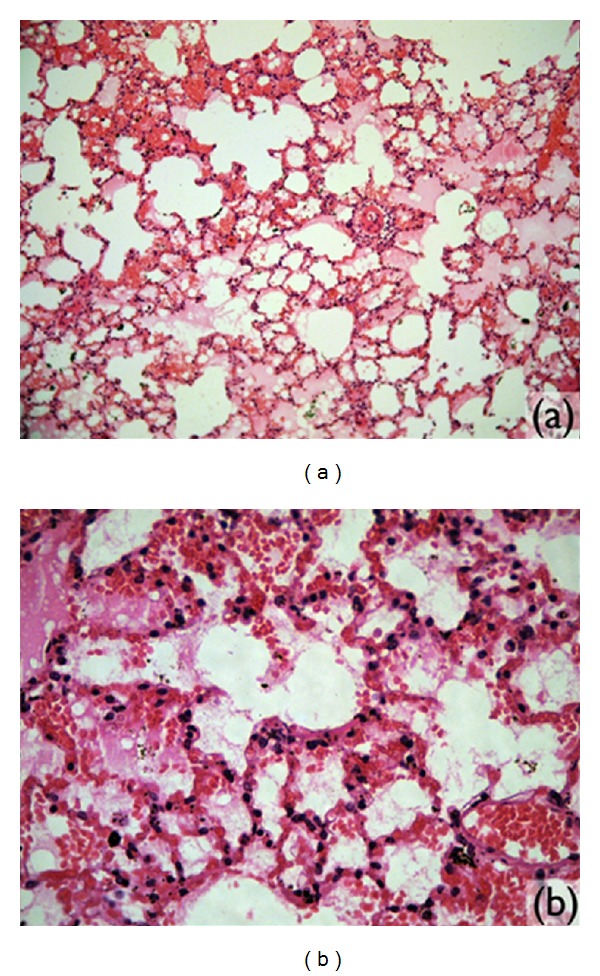
Microscopic images. (a) Lung tissue (magnified 10x). (b) Location of intraalveolar hemorrhaging (increase of 40x).

 In the lung sections, (Figure 6) small points of intra-alveolar hemorrhaging were found in three animals of the GB group. The rest of the lung tissue had normal histological standards. The capillary endothelium, basal membrane, interstices, alveolar epithelium, and alveolar macrophages had no alterations.

### 3.2. SD Group

**Figure 7:**
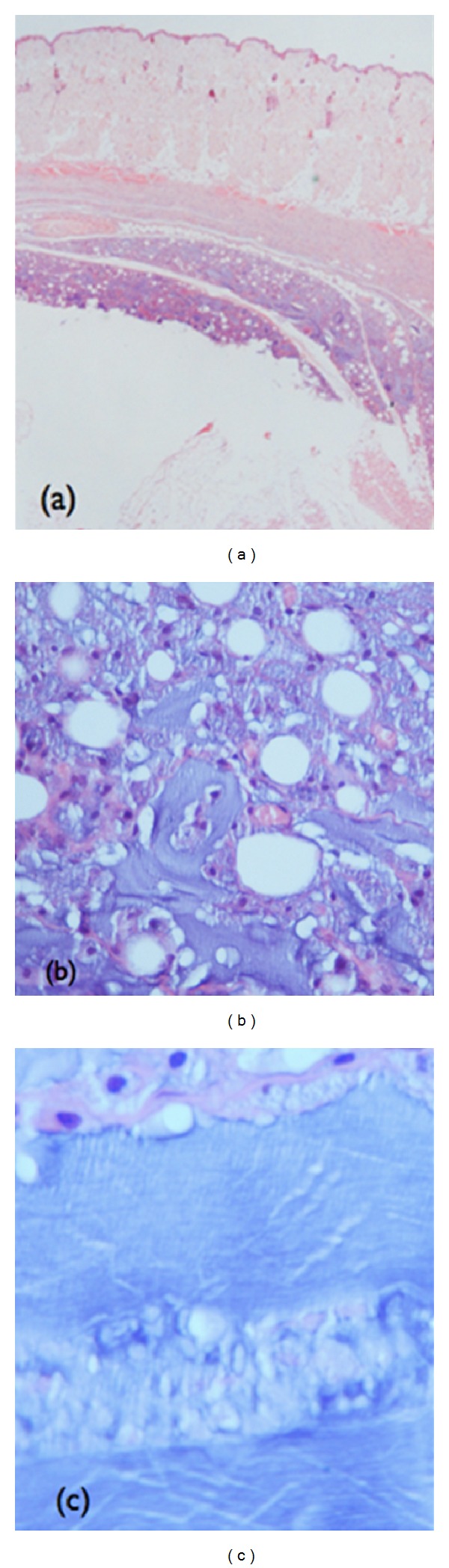
Histological section containing amorphous material found in the subcutaneous of one of the animals of the SD group. (a) Deposit of exogenous material enveloped by histiocytic reaction (magnified 2x). (b) Amorphous fibrillar material in the interstice and interior of the macrophages (magnified 40x). (c) Fibrillar aspect of exogenous material (magnified 100x).

In all the parts analyzed referred to the liver, spleen, and kidney, from this group, no significant alteration was found. A grayish material was identified on the slide of the subcutaneous cell tissue from one of the animals. The location contained exogenous material enveloped by a hystiocitic reaction. The substance presented fibrillar characteristics and was located in the interstice and interior of the macrophages (Figure 7). Microspheres or signs of fibrosis were not found, which are characteristics of PMMA implants already described in the literature [5, 6, 8, 10, 13, 14]. 

In the lungs, locations in the lung were found with foamy histiocytes in alveoli and intra-alveolar hemorrhaging. No inflammatory reaction or signs of pulmonary necrosis were found in the affected areas.

### 3.3. IP Group

**Figure 8:**
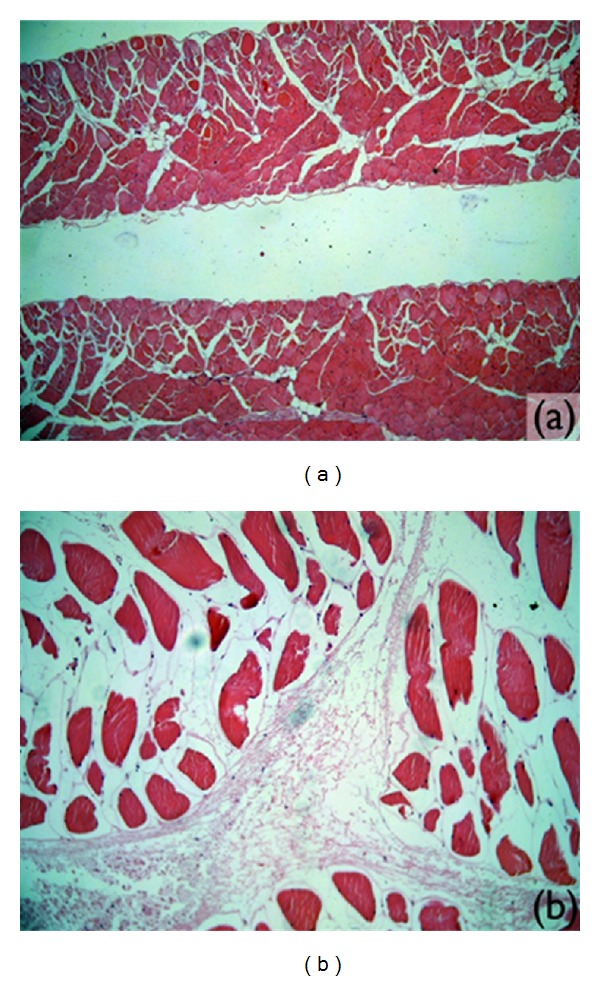
Microscopic images of muscle tissue without significant alterations. (a) Diaphragm (magnified 4x). (b) Rear right foot (magnified 10x).

No alteration was found in the histological sections of the diaphragms (Figure 8(a)), liver, kidney, and spleen analyzed. In two animals, congestion was observed in the histological analysis of the spleen, even if the material compatible with microspheres was not found in these organs. In all the sections containing the lung tissue of the animals in this group, locations of intra-alveolar hemorrhaging were observed. In one of these animals, these alterations were extensive and significant (Table 2).

### 3.4. MPD Group

None of the histological sections of the liver, kidney, and spleen had significant alterations. In the macroscopic analysis of the musculature, nodulations or signs of the PMMA implant were not found. The same was true in the microscopic analysis, where the musculature had a normal quality (Figure 8(b)), and no microspheres were found. In this group, pulmonary alterations were observed in three of the animals studied (Table 2). The alterations found in the distinct animals wereas follows:

- intra-alveolar hemorrhaging,
- intra-alveolar hemorrhaging with foamy hystocites,
- point of bronchial pneumonia.

None had signs of necrosis or significant inflammatory activity.

## 4. Discussion

The use of PMMA for dermal fillers has grown significantly in Brazil, as well as the number of complications described that are inherent to their use. Meanwhile, the quantity of studies and publications about the use of this substance as a dermal filler has not accompanied the speed of its use, not only in Brazil. Upon injection under the skin, PMMA provokes a tissue reaction with a formation of collagenous tissue and neovascularization around the microsphere [8, 11–15]. According to an experimental study in Wistar rats, the process would begin two weeks after the realization of the implants and continue for months [12]. 

In other studies, analyzing phagocytosis [15] and the permanence [5] of subdermal implants of Artecoll, the location of the implant containing PMMA was always easily located. In another histological study, where one of the authors applied different fillers in his own arm, the Artefill implants did not present alteration in the initial volume even after nine months of evolution, which suggests low or no degradation or migration of the product [5]. 

Lemperle et al. [6] describe three mechanisms by which PMMA microspheres can migrate.

- Hematogenic route, by inadvertent injection in a blood vessel, which can carry microspheres to the circulatory system. The most probable location of the final destination is the pulmonary capillaries.
- Lymphatic route, by injection in thick lymphatic vessels. The local lymph nodes and the lungs would be the most probable final destinations. These two routes, when directly touched by the product, can serve as an argument for the presence of microspheres in the lymphatic system and in organs such as the lungs or any other that serves to filter blood or the lymphatic system.
- Phagocytosis route microspheres phagocytized by macrophages can be absorbed at the location of the implant, later migrating to the local lymph nodes.

The literature consulted created an expectation that PMMA would be found in the histological sections from the locations of injection in the SD and MPD groups, where skin with subcutaneous cell tissue and musculature of the pelvic membrane, respectively, were analyzed. This immediately led to reasoning that by injecting the substance in the free peritoneal and knowing the mechanism for diaphragmatic absorption, there was a possibility of finding these spheres in the diaphragmatic windows. McClelland et al. [16], in a comparative study between Zyderm (collagen) and Artecoll applied in guinea pigs, showed that the product is susceptible to phagocytosis and degradation and can cause a local inflammatory response increasing the signs of immunogenicity of the PMMA particles. In this study, the guinea pigs also presented transepithelial elimination of the product, after intradermal application.

Capella [13], in an experimental study with intramuscular injections of Metacrill in the rear right leg of Wistar rats, observed alterations in local lymph nodes with histiocytes containing basophile granulocyte material in cytoplasm. In this study, the PMMA was identified in the histological sections from all of the 16 rats analyzed. Although all of these possibilities for possible migration were studied, the experiment did not verify this expectation because PMMA microspheres were not found in any of the locations inoculated and studied by posterior histopathology. The findings, thus, contrast with the literature studied.

Considering this information, four possibilities can be suggested to explain the fact that PMMA microspheres were not found in the location of injection in the SD and PD groups.

(i) The location chosen in the macroscopy for the assemblage of histological slides may not include the site of injection of the material.

This is not very likely given the experience of the pathologist and the ease of finding nodules formed by the injection of 0.2 mL of the substance, which indicates the need to assemble a new block of paraffin with the rest of the material and to conduct a study in series in sections of 4 micrometers.

(ii) The material may be absorbed by phagocytosis and enzymatic degradation.

(iii) The material may have migrated to other body parts even if they were not found in the filtering organs.

(iv) The implants contained only the vehicle of the product, and the microspheres remained in the syringe used for application.

The PMMA may have decanted in the syringe. The agitation of the material is not called for by the manufacturer and was not conducted before the injections. It is relevant that two animals of the PD group had splenetic alterations, which may be indications of migration of PMMA, by lymphatic means. Nevertheless, the single finding of nonspecific splenetic alterations, without PMMA microspheres, is not sufficient evidence to defend the hypothesis of migration of the inoculated substance.

Concerning the pulmonary alterations found, the intra-alveolar hemorrhaging found in 12 of the 16 animals is compatible with *post mortem* alterations, given that the animals were submited to painless death induced by saturation of ethyl ether, before having their organs removed for analysis. This can reveal alteration of the pulmonary parenchyma by the inhalation of supersaturated ethyl ether. The fact that the animals of all the groups had these alterations reinforces the theory that they were provoked during their deaths. Other alterations, such as points of bronchial pneumonia and foamy histiocytes, observed in two animals, are nonspecific alterations and may not be related to the application of PMMA but to the inhalation of ether.

Many of the published cases of complications with facial injections of PMMA related to dermal reactions such as erythema and telangiectasia are characterized as complications resulting from technical errors during the application, when the implants touch surface planes of the epidermis. There are also records of formation of granulomas of foreign bodies with vacuoles and giant cells, where the physiopathology is still not totally explained. However, it is known that a 2nd and 3rd application of the product can be related to severe infection or facial trauma [17]. Nevertheless, these skin alterations cannot be observed in rodents, because they do not have subcutaneous cell tissue.

The inadvertent injection in blood vessels also appears to be an important complication. Although there are few published cases, the morbidity of the consequences caused by this fact is quite high. There is a report of a case of ophthalmoplegia and total amaurosis after injection of PMMA in the glabellar region of a previously healthy patient [18]. Although puncture was tested in all of the inoculations with the return of blood to the syringe, in rats without subcutaneous tissue, the vascular network is certainly diminished, and there is a lower probability of one of these structures being punctured. In addition, the vascular volume of rodents of this weight is quite limited.

Most of the aesthetic facial implants of PMMA take place in deep reticular dermis and at the dermal-subdermal junction, where there is an intense venous and lymphatic network.

The diameter of the vessels of this region vary from 35 to 120 *μ*m (veins) and 30–100 *μ*m (lymphatic), which is sufficient to carry the PMMA microspheres. In the skin, especially that of the face, there is a network so dense that, theoretically, any puncture in this location winds up affecting one of these vessels [6]. Seeking to avoid vascular lesions, there are authors who defend the use of microcannulas instead of needles with sharp points [19]. 

In this study, hypodermic needles were used with sharp points and no sign of necrosis or vascular damage was found in the sections of the eyeball and others analyzed. No signs of rejection or extensive inflammatory process was found in any of the implants of the study.

In the bibliography studied, no studies were found that detail the exact composition and the real size of the microspheres found in the options for aesthetic facial filling sold in Brazil. It is important to have quality control of the PMMA substances, given that previous studies have shown that microspheres with diameters lower than 15 *μ*m are susceptible to phagocytosis and the impurities are related to exacerbated inflammatory reactions [6, 7, 20]. In Europe, publications such as that of McClelland et. al., which demonstrate irregularities in the composition of PMMA produced by Artes Medical, led to improvements in the substance with new purification processes. There are various studies that show the biocompatibility of PMMA. A perfect spherical form, the purity of the product and the neutrality of the electric charge are characteristics that cause the microspheres to resist phagocytosis [15]. 

Microspheres were not found in any of the sections made from the filtering organs in any of the animals studied. The analysis of only one section, which encompasses a small area of each organ, made it difficult to detect small microspheres of approximately 40 *μ*m in diameter. The results encountered were not in keeping with those cited in the literature. No signs of PMMA were found in any of the animals studied. Only one of the sections of SD had a nonidentified amorphous material not compatible with the characteristics already well described for PMMA implants.

Given that this is the first step of a study conducted by the Plastic Surgery Center of the University Hospital at UFSC, and the initiation of a line of research about the subject in our field, it is hoped that this study will help spark the curiosity of those who use the product, contributing to the scientific understanding of the behavior *in vivo* of implants of Polymethylmetacrylate available in Brazil. Therefore, the conclusion obtained in this experiment reveals that in the histopathological analysis of this work, no microspheres of PMMA were found in any of the tissues studied.

## Figures and Tables

**Table 1:** Groups division according to the location of the implant.

Group	Compartment	Local	Number of animals
GB	Intradermic	Glabella	4
SD	Subdermal	Cervical region	4
IP	Intraperitoneal	Abdomen	4
MPD	Intramuscular	Right leg	4

**Table 2:** Results found after histopathological analysis.

Group	N° animal	Organ location	Lung	Liver	Kidney	Spleen
GB	1	Normal	Normal	Normal	Normal	Normal
	2	Normal	A2^†^	Normal	Normal	Normal
	3	Normal	A2^†^	Normal	Normal	Normal
	4	Normal	A2^†^	Normal	Normal	Normal

SD	5	Normal	A3^‡^	Normal	Normal	Normal
	6	A1*	Normal	Normal	Normal	Normal
	7	Normal	A2^†^	Normal	Normal	Normal
	8	Normal	Normal	Normal	Normal	Normal

IP	9	Normal	A4^§^	Normal	Normal	Normal
	10	Normal	A2^†^	Normal	Normal	A6^¶^
	11	Normal	A2^†^	Normal	Normal	Normal
	12	Normal	A2^†^	Normal	Normal	A6^¶^

PD	13	Normal	A2^†^	Normal	Normal	Normal
	14	Normal	A3^‡^	Normal	Normal	Normal
	15	Normal	A5^||^	Normal	Normal	Normal
	16	Normal	Normal	Normal	Normal	Normal

A1*: presence of exogenous, fibrillar, and grayish material surrounded by hystiocytic reaction; A2^†^: small points of intra-alveolar hemorrhaging; A3^‡^: locations with foamy hystiocytes in alveolus; A4^§^: extensive and significant intra-alveolar hemorrhaging; A5^||^: locations of intra-alveolar hemorrhaging. +: areas with foamy hystiocytes in alveolus. +: point of bronchial pneumonia; A6^¶^: congested spleen.
